# Demonstration of *p*-type stack-channel ternary logic device using scalable DNTT patterning process

**DOI:** 10.1186/s40580-023-00362-w

**Published:** 2023-03-09

**Authors:** Yongsu Lee, Heejin Kwon, Seung-Mo Kim, Ho-In Lee, Kiyung Kim, Hae-Won Lee, So-Young Kim, Hyeon Jun Hwang, Byoung Hun Lee

**Affiliations:** Center for Semiconductor Technology Convergence, Department of Electrical Engineering, Pohang University of Science and Technology, Cheongam-Ro 77, Nam-Gu, Pohang, Gyeongbuk 37673 Republic of Korea

**Keywords:** Organic semiconductor, Photolithography, Ternary logic, Stack-channel, Zero differential conductance

## Abstract

A *p*-type ternary logic device with a stack-channel structure is demonstrated using an organic *p*-type semiconductor, dinaphtho[2,3-*b*:2',3'-*f*]thieno[3,2-*b*]thiophene (DNTT). A photolithography-based patterning process is developed to fabricate scaled electronic devices with complex organic semiconductor channel structures. Two layers of thin DNTT with a separation layer are fabricated via the low-temperature deposition process, and for the first time, *p*-type ternary logic switching characteristics exhibiting zero differential conductance in the intermediate current state are demonstrated. The stability of the DNTT stack-channel ternary logic switch device is confirmed by implementing a resistive-load ternary logic inverter circuit.

**Supplementary Information:**

The online version contains supplementary material available at 10.1186/s40580-023-00362-w.

## Introduction

Organic thin-film transistors (TFTs) have received considerable attention for application in next-generation electronics, such as wearable/stretchable devices [1–4], flexible electronics [5–7], electronic skin [8, 9], and monolithic three-dimensional integration circuits (M3DICs) [10, 11]. Recently, several studies have reported that organic semiconductors exhibit remarkable electrical performance, such as mobility in excess of 10 cm^2^/Vs and an on/off current ratio of approximately 10^10^ [7, 12–15]. Additionally, the leakage current of an organic TFT is lower than that of a polycrystalline-Si TFT, primarily owing to the large bandgaps of organic semiconductors (> 2.5 eV) [16, 17]. Moreover, the convenient low-temperature fabrication process of organic TFT is advantageous when implementing the M3DIC system.

Organic TFTs can be fabricated using various kinds of integration processes. Inkjet printing has been used to draw organic semiconductors and metal electrodes, and integrated circuits having dozens of organic TFTs with a sufficiently high device yield [10, 18, 19]. A continuous edge casting method using a solution-supplying blade has been used for mm-scale large-area growth of organic semiconductors with high-frequency band operations [20, 21]. A shadow mask process is a simple and low-cost process and is widely used for the organic TFT process, especially for the organic semiconductor isolation and metal patterning process. This process yields excellent device and integrated circuit performance [22–25].

While the shadow mask process is a reasonable approach for large-area organic TFT applications, it is difficult to fabricate highly integrated circuits due to the parasitic leakage current paths between the devices and the increased off current [26, 27]. Therefore, it is still worth pursuing the direct channel material patterning process applicable to organic TFT circuits.

A photolithography-based patterning process for organic semiconductor channels has been investigated. Höppner et al. developed organic TFTs with a channel width/length (*W*/*L*) ratio of 25 μm/10 μm via photolithography [28]. However, the photoresist (PR) used for the patterning process could not be removed because of damage during the PR removal process. Furthermore, ultraviolet (UV) radiation used in lithography degraded the organic channel materials. Therefore, a new patterning strategy must be developed to not only scale down the device dimensions but also minimize the damage to the organic semiconductor layer to maintain device performance.

Dinaphtho[2,3-*b*:2′,3′-*f*]thieno[3,2-*b*]thiophene (DNTT), a *p*-type organic semiconductor oligomer that is air-stable owing to its high ionization potential, was selected for the patterning study. DNTT can withstand chemical and thermal decomposition better than other organic semiconductors [16, 28–30].

In this work, we report a facile patterning process for DNTT channel and S/D electrodes based on photolithography with Au metal hardmask, as well as a device application. The electrical characteristics of lithographically patterned DNTT TFTs on a few microscales are comparable to those of DNTT TFTs fabricated via a shadow mask process. Furthermore, we demonstrate a process to form multilayer organic semiconductor channels for application in various device structures, such as vertically stacked memory and logic devices. More specifically, unique ternary logic switching characteristics exhibiting zero differential conductance (ZDC) at the intermediate current state are obtained from the stack-channel device comprising two DNTT layers. Using this device, a functional resistive-load ternary logic inverter circuit is demonstrated, confirming that the proposed methodology is applicable to more complex organic devices and circuits.

## Methods

**Fig. 1:**
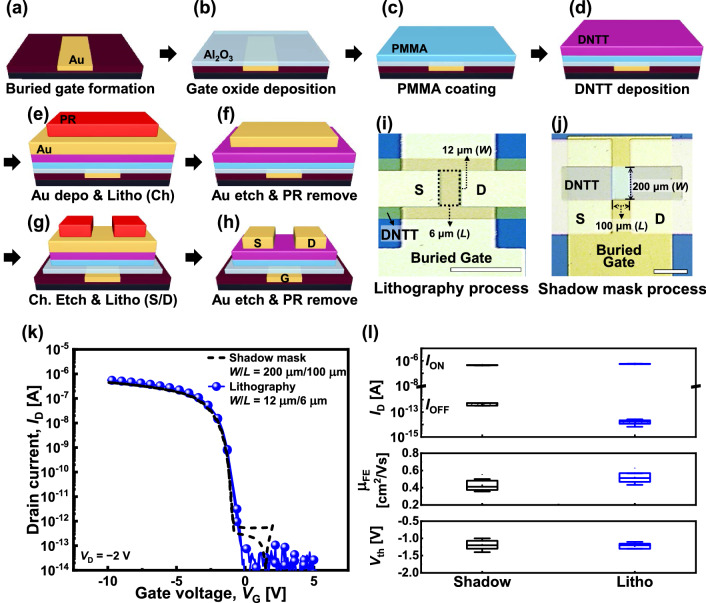
**a–h** Schematic of the fabrication process of the DNTT TFT based on the photolithography process. Photographs of the DNTT TFT fabricated via (**i**) photolithography and (**j**) shadow mask method, where *W*/*L* = 12 μm /6 μm and 200 μm /100 μm, respectively. Scale bars are 20 μm (**i**) and 200 μm (**f**). (**k**) *I*_D_–*V*_G_ curves of DNTT TFTs developed using different fabrication methods for *V*_D_ =  − 2 V. (**l**) Comparison of currents, field-effect mobility, and threshold voltage of the fabricated devices

To begin with, the SiO_2_/Si wafer was cleaned via sonication for 5 min in acetone, isopropyl alcohol, and distilled water in sequence. Subsequently, a 70 nm oxide trench was etched onto a 300 nm SiO_2_/Si wafer via photolithography and reactive ion etching with Ar and CF_4_ plasmas to form buried gate electrode patterns. While maintaining the PR for the lift-off process, a 10/60 nm Cr/Au metal layer was immediately deposited using an e-beam evaporator to fill the trench in a high vacuum chamber (~ 10^6^ Torr). Following the deposition, the buried gate electrode was formed by lift-off of the PR using acetone, which was followed by chemical–mechanical polishing of metal residues near the edge region of the gate pattern (Fig. 1a). As a gate dielectric, 10 nm of Al_2_O_3_ layer was deposited via atomic layer deposition (ALD) at 100 °C using trimethylaluminum (TMA) and H_2_O precursors. To improve the quality of the Al_2_O_3_ layer, an annealing process was performed at 300 °C in high vacuum (~ 10^−6^ Torr) (Fig. 1b). To reduce the hysteresis of the fabricated DNTT devices, 0–20 nm thick poly (methyl methacrylate) (PMMA, Sigma-Aldrich, Mw = 350,000) buffer layers were applied as a primer layer before the deposition of DNTT. The PMMA layer prevents the charging effect between the inorganic dielectric and DNTT [31]. To prepare the PMMA solution, PMMA powder was dissolved in toluene solvent, and the solution was stirred at 70 °C overnight to completely dissolve the PMMA powder. Thereafter, the PMMA solution was coated on top of the Al_2_O_3_ layer by spin-coating at 3000 rpm for 60 s. Subsequently, the solution was baked at 70 °C for 10 min to remove the remaining solvent (Fig. 1c). The thickness of the PMMA layer was modulated by varying the concentration of the PMMA solution. The thickness of the PMMA layer was measured using an ellipsometer and via atomic force microscopy (AFM). Following the PMMA coating, a 40 nm thick DNTT layer (Sigma-Aldrich) was thermally deposited in high vacuum, followed by baking at 130 °C for 20 min in ambient air (Fig. 1d). The PMMA layer became more resistant to PR removal during the high-temperature baking process (Additional file 1: Fig. S1).

For the DNTT/separation layer (SL) device and DNTT ternary logic device, a 1.5 nm Al_2_O_3_ layer was deposited onto the DNTT layer via ALD, followed by 0–20 nm of PMMA coating. Subsequently, the second 40 nm DNTT channel layer was additionally deposited for the DNTT ternary logic device.

For all the devices, the organic stack structures (DNTT, DNTT/SL, and DNTT ternary logic device) were simultaneously patterned. For channel patterning, a 30 nm Au hardmask was deposited via a thermal evaporation process. Contact photolithography was performed with a minimum critical dimension of ~ 2 µm (Fig. 1e). The exposed Au regions were etched using a gold etchant TFA (Transene) at 25 °C for 10 s. Thereafter, the PR mask was completely removed using dimethyl sulfoxide (DMSO) (Fig. 1f). Subsequently, the DNTT channel pattern was etched with an Au hardmask pattern via an oxygen plasma etching process (RF power = 50 W, O_2_ pressure = 350 mTorr). Here, the Au hardmask blocks UV light during photolithography and plasma processes [32], preventing degradation caused by the positive threshold voltage (*V*_th_) shift [33]. Subsequently, 70 nm of Au was blanket-deposited. Finally, another photolithography technique was applied to form source/drain (S/D) electrode patterns (Fig. 1g). Using the PR mask, S/D electrode patterns were formed via the Au wet etching process for 30 s, followed by PR mask removal (Fig. 1h). At the end of each step, the results of the process were examined using an optical microscope to ensure the robustness of the pattern (Additional file 1: Fig. S2).

As a reference, another DNTT TFT device with shadow masks that did not go through any patterning or etching processes of the DNTT channel and S/D electrodes was fabricated. The buried gate electrode, Al_2_O_3_ dielectric, and PMMA buffer layer were formed on SiO_2_/Si substrate, which is exactly the same as the process described above. Then the DNTT channel layer was deposited using a shadow mask and baked at 130 °C for 20 min. Lastly, 100 nm of Au was deposited using another shadow mask to form S/D electrodes. The *W* and *L* of the device fabricated via the lithography process were 12 and 6 μm, respectively (Fig. 1i), whereas those of the devices fabricated using shadow masks were 200 and 100 μm, respectively (Fig. 1j).

The final devices were electrically characterized using a semiconductor parameter analyzer (Keithley 4200) at room temperature under ambient air conditions.

## Results and discussion

Figure 1(a–h) present the fabrication process of DNTT TFT. General contact lithography was employed to fabricate the scaled organic semiconductor TFT, and an Au hardmask was introduced to minimize damage to the DNTT channel. The fabrication process is described in detail under the Methods section. Figure 1i illustrates the scaled DNTT TFT using the patterning process. Compared to the DNTT TFT using shadow masks (Fig. 1j), the dimensions of the scaled device are 17 times lower. Figure 1k illustrates the drain current–gate voltage (*I*_D_–*V*_G_) curves of the DNTT TFTs fabricated via lithography and the shadow mask process. The *W*/*L* ratios of the two devices are almost equal at ~ 2, and both are long-channel devices. Therefore, the transfer curves of the two devices almost overlap.

The electrical properties of on current (*I*_ON_), off current (*I*_OFF_), field-effect mobility (*μ*_FE_), and *V*_th_ for shadow mask and lithography TFTs were examined, as depicted in Fig. 1l. For 25 devices, *I*_ON_, *I*_OFF_, *μ*_FE_, and *V*_th_ are 5.74 ± 0.19 × 10^−7^ A, 1.63 ± 0.51 × 10^−14^ A, 0.52 ± 0.05 cm^2^/Vs, and − 1.11 ± 0.09 V for lithography devices, and 4.44 ± 0.16 × 10^−7^ A, 4.30 ± 1.06 × 10^−13^ A, 0.43 ± 0.05 cm^2^/Vs, and − 1.18 ± 0.11 V for shadow mask devices, respectively. Compared to the shadow mask device that did not undergo PR coating and UV exposure, the electrical characteristics of the lithographically patterned DNTT TFTs were seldom degraded. In particular, *I*_OFF_ was lower because the overlap area between the S/D electrodes and the buried gate was smaller (1.0 × 10^4^ μm^2^ versus 1.1 × 10^3^ μm^2^).

**Fig. 2:**
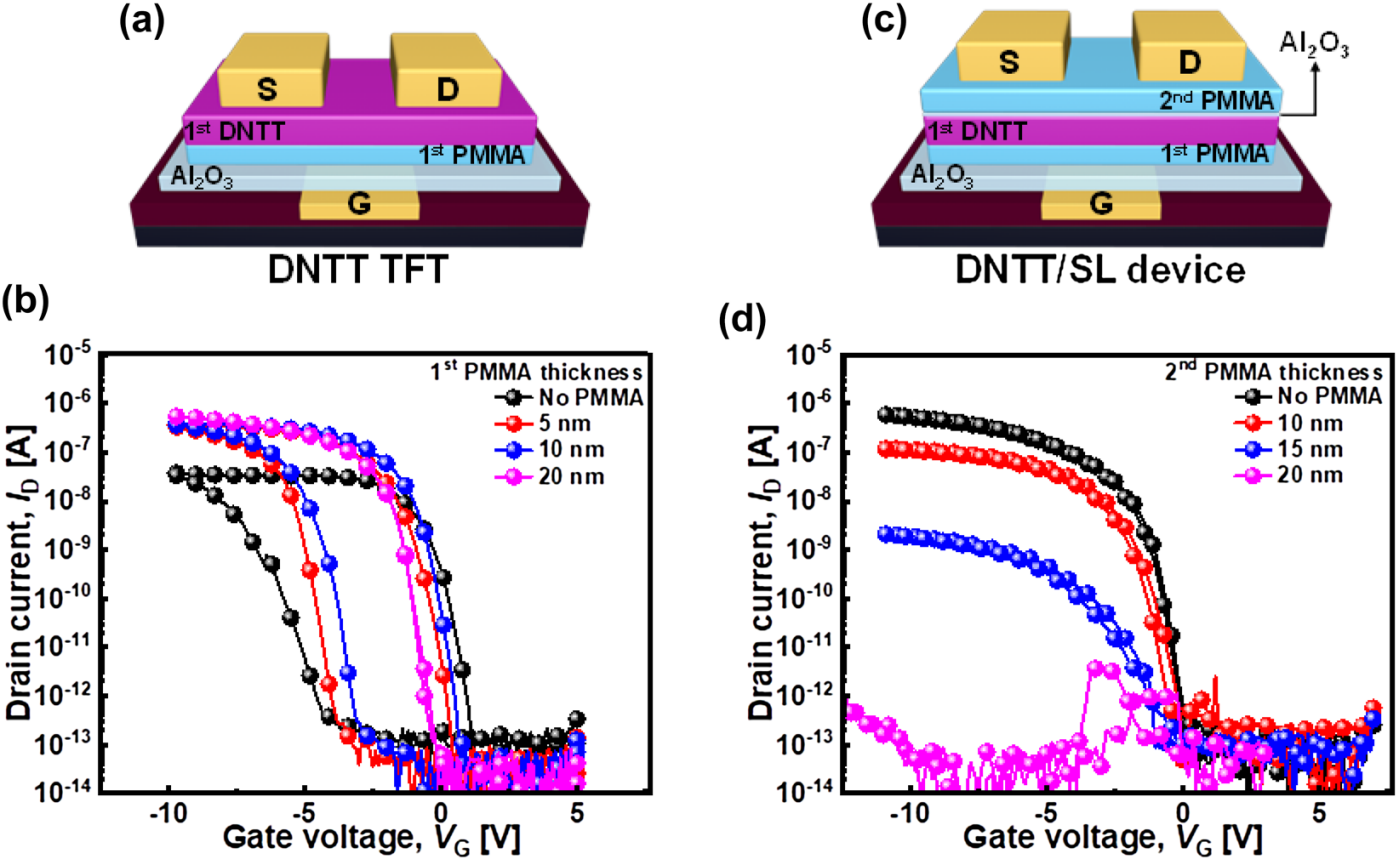
**a** Schematic of the device structure. **b** Electrical characteristics of the DNTT TFT with various thicknesses of the first PMMA layer for *V*_D_ =  − 2 V. **c** Schematic of the device structure.**d** Electrical characteristics of the DNTT/SL device with various thickness of the second PMMA layer for *V*_D_ =  − 2 V, where the thickness of the first PMMA is 20 nm

Figure 2a and b display a schematic of the device structure and the transfer characteristics of the DNTT TFT modulated by the thickness of the PMMA layer deposited prior to the DNTT deposition. As mentioned in the Methods section, the first PMMA layer reduced the hysteresis of DNTT TFTs. Without the PMMA buffer layer, the hysteresis was extremely large, and *I*_ON_ degraded to 10^−8^–10^−7^ A, primarily because of the strong charge exchange effect at the interface between the gate dielectric and DNTT channel [31]. However, the hysteresis decreased as the thickness of the PMMA layer increased and became negligible at a PMMA thickness of 20 nm. However, the disadvantage of using a thick PMMA buffer layer is the decrease in the gate capacitance.

Figure 2c and d depict a schematic of the device structure and the transfer characteristics of the DNTT TFT with a SL on top of the channel region (DNTT/SL device). In these devices, 1.5 nm of the Al_2_O_3_ layer was deposited to protect the underlying DNTT layer, and the second PMMA layer was sequentially coated while varying the thickness of the second PMMA layer from 0 to 20 nm. Here, the Al_2_O_3_/PMMA layer is denoted as SL because it separates the first and second DNTT layers in the stack-channel DNTT device to be described later and shields the first DNTT layer during the subsequent processes. The thickness of the first PMMA layer was fixed at 20 nm.

The second PMMA layer acted as the primary layer for the second DNTT layer. As the thickness of the second PMMA layer increased, the drive current gradually decreased because the SLs acted as a resistance layer between the S/D electrodes and DNTT channel. Owing to low hysteresis, the second PMMA layer primarily functioned as an insulation layer. However, even for the 20 nm PMMA case, the current increased as *V*_G_ exceeded − 8 V, owing to the field-induced leakage current.

Subsequently, the second DNTT channel layer was added on top of the device structure depicted in Fig. 2c, particularly considering that the multiple-stack-channel structure can be used for diverse applications, such as vertical channel memory or logic devices. Lee et al. reported that a unique *n*-type ternary logic device can be achieved using a ZnO stack-channel [36].

A ternary logic device has three current states, representing three logic states (0, 1, and 2). It is well-known that ternary logic can perform the same logic functions with fewer devices and shorter interconnect lengths than binary logic [34–40]. Thus, the complexity of the integrated circuits is reduced. Moreover, power consumption can be reduced.

**Fig. 3:**
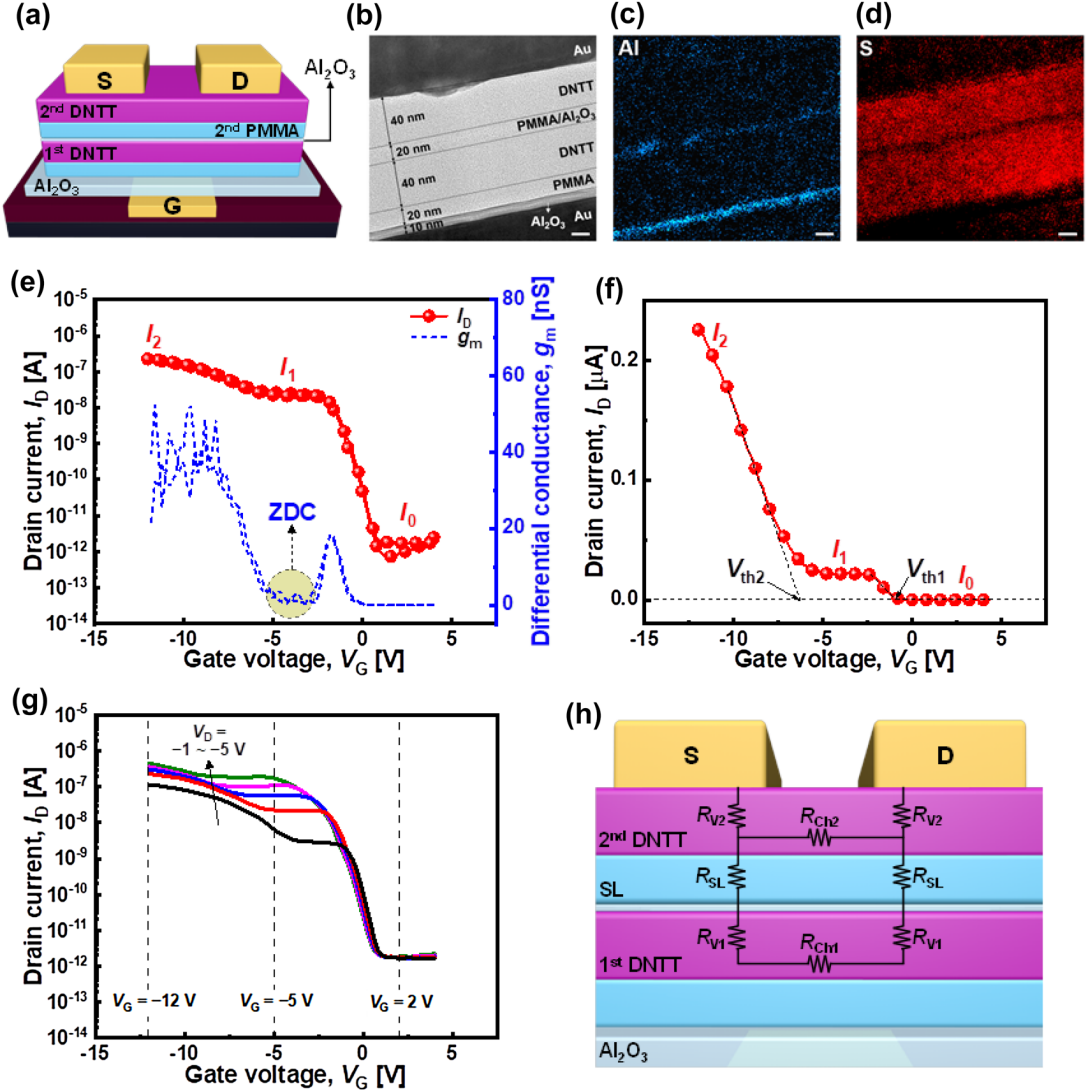
**a** Schematic of the DNTT ternary logic device structure. **b** Cross-sectional TEM image of the stack-channel ternary logic device and EDS analysis of the device for **c** Al and **d** S. Scale bar = 20 nm. **e** *I*_D_–*V*_G_ curve and differential conductance (*g*_m_) of the DNTT ternary logic device for *V*_D_ =  − 2 V. **f** *I*_D_–*V*_G_ curve of the device with a linear scale for *V*_D_ =  − 2 V. *V*_th_ for *I*_1_ and *I*_2_ (*V*_th1_ and *V*_th2_, respectively) are defined on the linear scale transfer curve via the linear extrapolation method. **g** *I*_D_–*V*_G_ curve of the device for *V*_D_ =  − 1 to − 5 V in − 1 V steps. **h** Cross-sectional schematic of the device structure of the DNTT ternary logic device with various resistance components

Figure 3a illustrates the schematic of the DNTT ternary logic device with a stack-channel structure comprising the first PMMA layer/first DNTT layer/SL (Al_2_O_3_/second PMMA layer)/second DNTT layer. The fabrication process is described in detail under the Methods section (and Additional file 1: Fig. S4). Figure 3b illustrates the cross-section of the transmission electron microscope (TEM) image of the channel stack, in which the Al_2_O_3_ gate dielectric, Au gate electrode, and Au S/D electrodes can be easily identified. However, DNTT and PMMA are difficult to distinguish because they are carbon-based materials with amorphous structures. Energy dispersive spectroscopy (EDS) analysis was performed to determine the layer structure. Figure 3c illustrates the EDS mapping of the aluminum atoms, showing the gate dielectric and SL. Figure 3d presents the EDS mapping of the sulfur atoms. The EDS mapping exhibits two DNTT layers separated by the SL because DNTT has sulfur atoms in its molecular structure (C_22_H_12_S_2_).

Figure 3e presents the *I*_D_–*V*_G_ curves of the DNTT ternary logic device. As described previously, with suitably optimized PMMA layers, the hysteresis was almost negligible. Furthermore, the transfer curve exhibited typical ternary logic device characteristics for three current states: *I*_0_, *I*_1_, and *I*_2_. The off and on currents were defined as *I*_0_ and *I*_2_, respectively, while the flat saturated current between *I*_0_ and *I*_2_ was defined as *I*_1_. Remarkably, the portion of the intermediate current (*I*_1_) exhibited an almost flat current region independent of *V*_G_ that is comparable to the previously reported results for *n*-type ZnO stack-channel ternary logic switch devices. This behavior was named as the ZDC characteristic owing to the extremely small slope of the transfer curve in the intermediate state. The unique ZDC characteristic is further evident on a linear scale, as shown in Fig. 3f. The presence of the ZDC region is crucial for the circuit application of ternary logic switch devices because it can improve the noise margin of ternary logic circuits, which is a critical weakness of ternary logic technology.

Figure 3g exhibits the drain voltage (*V*_D_) bias dependence of the transfer curves. More specifically, as |*V*_D_| increased, the off current, *I*_0_, hardly changed, whereas both *I*_1_ and *I*_2_ were modulated by |*V*_D_|. In the present work, it was found that *I*_1_ was more strongly influenced by |*V*_D_| than *I*_2_, which is related to the structure and operational mechanism of the *p*-type stack-channel device. Figure 3h displays the cross-sectional schematic of the DNTT ternary logic device structure, illustrating resistance components in the stack-channel structure. According to the operation mechanism, the top channel (second DNTT layer) is first turned on (in the low |*V*_G_| region), and subsequently the bottom channel is turned on (in the high |*V*_G_| region) (Additional file 1: Fig. S6). Thus, the top channel is the primary source of the intermediate current *I*_1_. More specifically, *I*_1_ is determined by 2*R*_V2_ + *R*_Ch2_, where R_V2_ and R_Ch2_ denote the vertical and channel resistances of the second DNTT channel. In this case, *I*_1_ is more strongly influenced by |*V*_D_|. Meanwhile, *I*_2_ is less dependent on |*V*_D_| because of the additional series resistance components from the SL and first DNTT channels, expressed as 2(*R*_V2_ + *R*_SL_ + *R*_V1_) + *R*_Ch1_, where *R*_SL_ denotes the resistance of the SL, and *R*_V1_ and *R*_Ch1_ denote the vertical and channel resistances of the first DNTT channel, respectively. Note that the contact resistances between the S/D electrodes and second DNTT channel are ignored for simplicity.

**Fig. 4:**
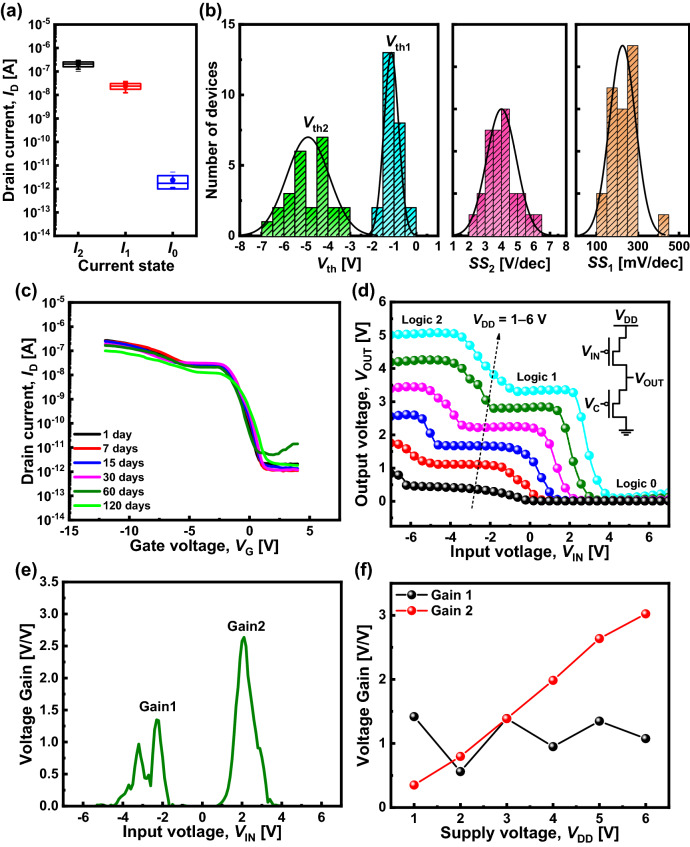
**a** Statistics of the current level of each state as *I*_2_ (on state), *I*_1_ (intermediate state), and *I*_0_ (off state). **b** Histograms of the threshold voltages and subthreshold swings of 25 separate DNTT ternary logic devices for *V*_D_ = − 2 V. **c** Electrical characteristics of the DNTT ternary logic device intermittently measured for an extended period of over 120 days to investigate long-term stability for *V*_D_ =  − 2 V. **d** VTCs of the ternary logic inverter for *V*_DD_ = 1–6 V. Inset: schematic of a resistive-load ternary logic inverter. **e** Voltage gains of the two-state transition for *V*_DD_ = 5 V. **f** Voltage gains versus *V*_DD_

Prior to circuit-level fabrication, the stability and uniformity of the DNTT devices were examined using 25 devices fabricated on the same wafer. Figure 4a depicts the relatively narrow distributions of *I*_2_, *I*_1_, and *I*_0_: 2.06 ± 0.48 × 10^−7^, 2.40 ± 0.64 × 10^−8^, and 2.34 ± 1.34 × 10^−12^ A, respectively. For ternary logic circuit demonstration, the uniformity of *I*_1_ is the most crucial factor because the level of *I*_1_ should be matched among the ternary logic devices. The distributions of *V*_th_ and subthreshold swing (*SS*) are depicted in Fig. 4b, where *V*_th1_ and *SS*_1_ refer to the threshold voltage and subthreshold swing at the first transition (*I*_0_–*I*_1_), and *V*_th2_ and *SS*_2_ refer to those at the second transition (*I*_1_–*I*_2_), respectively. *V*_th1_ and *SS*_1_ exhibited reasonably tight distributions (− 1.1 ± 0.3 V and 226 ± 63 mV/dec) for the lab-scale device process.

Figure 4c depicts the degradation of the monitored transfer characteristics, in which the ternary logic functionality of the DNTT ternary logic device is effectively maintained over an extended test period. Specifically, the intermediate current level (*I*_1_) is a critical electrical characteristic in ternary logic circuits. If the degradation criterion is set at 15%, the device is operable for 64 days. To further improve air stability, a proper passivation process should be investigated [41].

Finally, a resistive-load ternary logic inverter (shown in the inset of Fig. 4d) was implemented to demonstrate the practicality of the DNTT-based ternary logic device. The circuit uses two DNTT ternary logic devices; however, the device on the pull-down network connected to the ground bias is used as a resistive-load by applying a constant gate bias. Instead of using a resistor in the pull-down network, the resistance from the saturated current region of the ternary logic device automatically matches the resistance of the pull-up network, representing an intermediate state of the inverter for all supply voltage (*V*_DD_).

The voltage transfer characteristics (VTCs) of the ternary logic inverter displayed in Fig. 4d confirm the successful operation of the ternary logic inverter. For *V*_DD_ = 1–6 V, all VTCs of the ternary logic inverter exhibited three states: logic 0, 1, and 2. More specifically, logic 0 converged to the ground level (0 V), and a flat logic 1 was presented at half the* V*_DD_ level. A stable state of logic 2 was also represented; however, it did not fully reach the *V*_DD_ level; only 83–87% of *V*_DD_ was obtained. This can be improved by enhancing the *I*_2_/*I*_1_ current ratio of the ternary logic device, such that *V*_DD_ from the power supply is applied to the pull-up network for logic 2. Figure 4e presents the typical voltage gains as a function of *V*_IN_. Two peaks of voltage gains were obtained from the transitions between adjacent logics. Figure 4f illustrates the voltage gains extracted as a function of *V*_DD_. Gain 2 was proportional to *V*_DD_, whereas Gain 1 was independent of *V*_DD_ and lower than Gain 2. The performance of the ternary logic circuits can be further improved by configuring a complementary ternary logic circuit using *n*- and *p*-type stack-channel ternary logic devices.

## Conclusion

A facile integration process incorporating photolithography-based patterning was developed to fabricate scaled devices with a single-layer or double-layer organic semiconductor (DNTT) channel. The *p*-type ternary logic switch device with a double-layer stack-channel structure exhibited an intermediate current state with a unique ZDC region. Moreover, a resistive-load ternary logic inverter combining two *p*-type ternary logic switch devices was implemented. The results demonstrated that the *p*-type ternary logic device exhibits robust stability by sustaining the subsequent integration process and long-term air exposure, confirming that the photolithography-based patterning process is applicable to complex organic device applications, such as memory and logic systems.

## Supplementary Information

**Additional file 1: Fig. S1**. Photographs of the 20 nm PMMA layer on 2 cm × 2 cm Si/SiO_2_ wafers following half dipping in DMSO at different PMMA baking temperatures of (**a**) 70 ℃ and (**b**) 130 ℃. Scale bar = 100 nm. **Fig. S2**. Photographs of the fabrication process of DNTT TFT via photolithography, scale bar = 10 μm. **Fig. S3**. Histograms of (**a**) on and off currents, (**b**) field-effect mobility, (**c**) threshold voltage, and (**d**) subthreshold swing of 25 separate DNTT TFT devices for *V*_D_ =  − 2 V. **Fig. S4**. (**a**–**h**) Schematic of the fabrication process flow of the DNTT/SL device and DNTT ternary logic device. **Fig. S5**. (**a**) Schematic of the device structure and (**b**) electrical characteristics of the full-stack devices with a 10 and 15 nm second PMMA layer for *V*_D_ =  − 2 V, where the thickness of the first PMMA layer is 20 nm. **Fig. S6**. (**a**) Electrical characteristics of the DNTT ternary logic device for different operation regions. Expected operation mechanisms of the device in (**b**) Region I (*V*_th1_ < *V*_G_), (**c**) Region II (*V*_th2_ < *V*_G_ < *V*_th1_), and (**d**) Region III (*V*_G_ < *V*_th2_).

## Data Availability

The datasets used and/or analyzed during the current study are available from the corresponding author on reasonable request.

## Abbreviations

DNTT: Dinaphtho[2,3-*b*:2',3'-*f*]thieno[3,2-*b*]thiophene
TFT: Thin-film transistor
M3DIC: Monolithic three-dimensional integration circuit
*W*/*L*: Width/length
PR: Photoresist
UV: Ultraviolet
ZDC: Zero differential conductance
ALD: Atomic layer deposition
TMA: Trimethylaluminum
PMMA: Poly(methyl methacrylate
AFM: Atomic force microscopy
SL: Separation layer
DMSO: Dimethyl sulfoxide
*V*_th_: Threshold voltage
S/D: Source/Drain
*I*_D_–*V*_G_: Drain current–gate voltage
*I*_ON_: On current
*I*_OFF_: Off current
*μ*_FE_: Field-effect mobility
TEM: Transmission electron microscope
EDS: Energy dispersive spectroscopy
*I*_0_: Off current
*I*_1_: Intermediate current
*I*_2_: On current
*V*_D_: Drain voltage
*SS*: Subthreshold swing
*V*_DD_: Supply voltage
VTC: Voltage transfer characteristics
