# Supplementary material for: Demonstration of p-type stack-channel ternary logic device using scalable DNTT patterning process
Source: Nano Converg. 2023 Mar 9;10:12. doi: 10.1186/s40580-023-00362-w (PMC9998751; doi:10.1186/s40580-023-00362-w)
Supplement: Supplementary file 1 — Additional file 1: Fig. S1. Photographs of the 20 nm PMMA layer on 2 cm × 2 cm Si/SiO2 wafers following half dipping in DMSO at different PMMA baking temperatures of (a) 70 ℃ and (b) 130 ℃. Scale bar = 100 nm. Fig. S2. Photographs of the fabrication process of DNTT TFT via photolithography, scale bar = 10 μm. Fig. S3. Histograms of (a) on and off currents, (b) field-effect mobility, (c) threshold voltage, and (d) subthreshold swing of 25 separate DNTT TFT devices for VD = − 2 V. Fig. S4. (a–h) Schematic of the fabrication process flow of the DNTT/SL device and DNTT ternary logic device. Fig. S5. (a) Schematic of the device structure and (b) electrical characteristics of the full-stack devices with a 10 and 15 nm second PMMA layer for VD = − 2 V, where the thickness of the first PMMA layer is 20 nm. Fig. S6. (a) Electrical characteristics of the DNTT ternary logic device for different operation regions. Expected operation mechanisms of the device in (b) Region I (Vth1 < VG), (c) Region II (Vth2 < VG < Vth1), and (d) Region III (VG < Vth2). [file 40580_2023_362_MOESM1_ESM.docx]

Additional file 1 Information

**Demonstration of *p*-type stack-channel ternary logic device using scalable DNTT patterning process**

Yongsu Lee, Heejin Kwon, Seung-Mo Kim, Ho-In Lee, Kiyung Kim, Hae-Won Lee, So-Young Kim, Hyeon Jun Hwang*, and Byoung Hun Lee*

Center for Semiconductor Technology Convergence, Department of Electrical Engineering, Pohang University of Science and Technology, Cheongam-ro 77, Nam-gu, Pohang, Gyeongbuk 37673, Republic of Korea

*E-mail: hhjune@postech.ac.kr, bhlee1@postech.ac.kr

**Fig. S1**. Photographs of the 20 nm PMMA layer on 2 cm × 2 cm Si/SiO_2_ wafers following half dipping in DMSO at different PMMA baking temperatures of (**a**) 70 ℃ and (**b**) 130 ℃. Scale bar = 100 nm.

Figure S1 displays the photographs of the PMMA layer on 2 cm × 2 cm Si/SiO_2_ wafers at different baking temperatures. Upon coating the wafers with 20 nm PMMA layers, half of the wafers were dipped in dimethyl sulfoxide (DMSO, PR remover) for 30 s. First, the PMMA baked at 70 ℃ was etched by DMSO (Fig. S1(a)). However, the PMMA baked at 130 ℃ was left unetched, and there was no physical damage to the PMMA layer (Fig. S1(b)). This is because high-temperature annealing causes the PMMA polymers to cross-link with each other, which makes them chemically resistant.


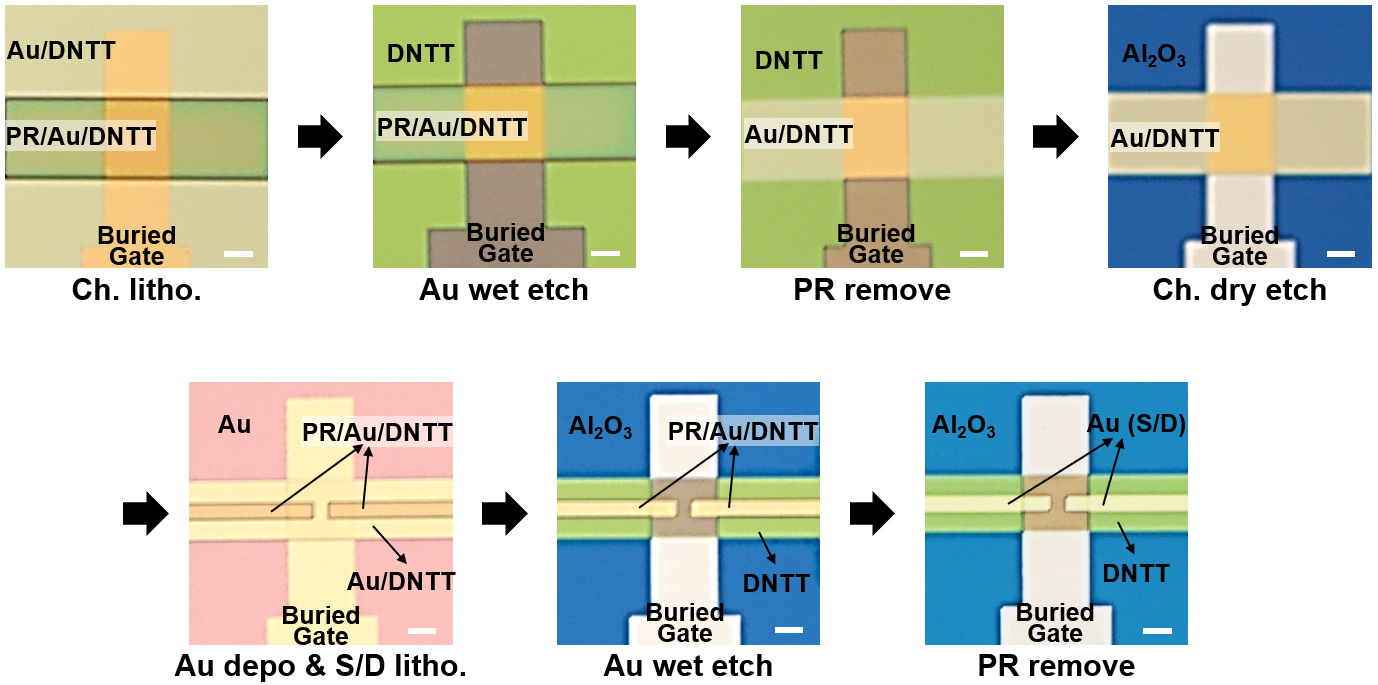


**Fig. S2**. Photographs of the fabrication process of DNTT TFT via photolithography, scale bar = 10 μm.

Figure S2 presents photographs of the fabrication process of the DNTT TFT via photolithography. Although various solutions, such as PR developer, metal etchant, and PR remover, were used in most of the fabrication processes, it was confirmed that there was no loss or damage to the DNTT, PMMA, and metal layers.

**Fig. S3**. Histograms of (**a**) on and off currents, (**b**) field-effect mobility, (**c**) threshold voltage, and (**d**) subthreshold swing of 25 separate DNTT TFT devices for *V*_D_ = −2 V.

Figure S3 illustrates the histograms of the electrical properties of the DNTT TFT fabricated via photolithography, which was examined using 25 devices. All electrical properties reveal tight distributions, where *I*_ON_ is 5.74 ± 0.19 × 10^−7^ A, *I*_OFF_ is 1.63 ± 0.51 × 10^−14^ A, *μ*_FE_ is 0.52 ± 0.05 cm^2^/Vs, *SS* is 242 ± 18 mV/dec, and *V*_th_ is −1.11 ± 0.09 V. The values of *I*_ON_, *I*_OFF_, *μ*_FE_, and *V*_th_ of Fig. 1(l) are extracted from Fig. S3.

**Fig. S4**. (**a**–**h**) Schematic of the fabrication process flow of the DNTT/SL device and DNTT ternary logic device.

Figure S4 shows the fabrication process of the DNTT/SL device and DNTT ternary logic device. The process of the DNTT/SL device fabrication is identical to that of the DNTT TFT until the first DNTT (Fig. 1(d)); the 1.5 nm Al_2_O_3_ and second PMMA layers were additionally deposited (Fig. S4(a)). For DNTT ternary logic device, the second DNTT layer was additionally deposited on the second PMMA layer (Fig. S4(e)). Subsequently, both stack layers were covered by a 30 nm Au hard mask (Fig. S4(b) and (f)), and the channel (Fig. S4(c) and (g)) and S/D electrodes were patterned via photolithography (Fig. S4(d) and (h)).

**Fig. S5**. (**a**) Schematic of the device structure and (**b**) electrical characteristics of the full-stack devices with a 10 and 15 nm second PMMA layer for *V*_D_ = −2 V, where the thickness of the first PMMA layer is 20 nm.

Figure S5(a) displays the device structure in which two DNTT channels are stacked, and the second PMMA layer is thinner than the case of Fig. 3(a), where the thickness of the first PMMA layer is the same (20 nm). Figure S5(b) illustrates the *I*_D_–*V*_G_ curves of the device with the structure shown in Fig. S5(a), where the thicknesses of the second PMMA are 10 and 15 nm. Unlike the electrical characteristics of the device with a 20 nm second PMMA layer (Fig. 3(e)), ternary logic characteristics were not observed. As shown in Fig. 2(d), the 10–15 nm thick second PMMA layer cannot block the current from the first DNTT channel; therefore, both currents from the first and second DNTT channels contribute to the total current for all *V*_G_ values. In other words, the ternary logic properties of the DNTT ternary logic device were obtained only when the second PMMA layer blocked the current of the first DNTT channel. Based on these observations, the expected operation mechanism of the DNTT ternary logic device is illustrated in Fig. S6.

**Fig. S6**. (**a**) Electrical characteristics of the DNTT ternary logic device for different operation regions. Expected operation mechanisms of the device in (**b**) Region Ⅰ (*V*_th1_ < *V*_G_), (**c**) Region Ⅱ (*V*_th2_ < *V*_G_ < *V*_th1_), and (**d**) Region Ⅲ (*V*_G_ < *V*_th2_).

Figure S6 illustrates the expected operation mechanisms of the DNTT ternary logic device. Figure S6(a) presents the typical electrical characteristics of the DNTT ternary logic device, in which the operation regions are divided into *V*_th1_ and *V*_th2_. The three operation regions, Ⅰ, Ⅱ, and Ⅲ correspond to the current states *I*_0_, *I*_1_, and *I*_2_, respectively. When *V*_G_ > *V*_th1_ (Region I), the second channel operates first and the current starts to increase from the initial current state (*I*_0_). As discussed in Fig. S5, it is expected that the first channel does not contribute to the current. As *V*_G_ exceeds *V*_th1_ (Region Ⅱ), the current continues to increase and subsequently saturates at *I*_1_. This is expected because hole charge carriers are induced in the first channel, and they screen the gate field that transfers them to the second channel. Finally, as *V*_G_ exceeds *V*_th2_ (Region Ⅲ), the carriers induced in the first channel move to the drain electrode by tunneling through the SL, thereby increasing the current (*I*_2_).
